# Molecular Characterization and Feeding-Associated Expression Dynamics of the Period Gene Family in Channel Catfish (*Ictalurus punctatus*)

**DOI:** 10.3390/cimb47060438

**Published:** 2025-06-09

**Authors:** Hongyan Liu, Shiyong Zhang, Xiaohui Chen, Minghua Wang, Liqiang Zhong, Yongqiang Duan, Bingjie Xie, Luyu Tang, Yi Cheng

**Affiliations:** 1 National Genetic Breeding Center of Channel Catfish, Freshwater Fisheries Research Institute of Jiangsu Province, Nanjing 210017, China; 2 The Jiangsu Provincial Platform for Conservation and Utilization of Agricultural Germplasm, Nanjing 210014, China; 3 College of Fisheries and Life Science, Shanghai Ocean University, Shanghai 201308, China; 4 College of Marine Science and Fisheries, Jiangsu Ocean University, Lianyungang 222005, China

**Keywords:** period gene family, channel catfish, circadian rhythm, feeding rhythm, gene expression

## Abstract

Rhythms, regulated by core clock genes like the period (*per*) gene family, are crucial for maintaining physiological processes in animals. In teleost fish, including channel catfish (*Ictalurus punctatus*), these genes have evolved distinct functions. However, the evolutionary characteristics and functional roles of period genes, particularly in response to environmental cues such as feeding, remain unclear. This study aimed to investigate the evolutionary divergence and functional specialization of the period gene family in channel catfish, with a focus on feeding-induced rhythmicity. Four period genes, *Ipper1b*, *Ipper2*, *Ipper2l*, and *Ipper3*, were identified in channel catfish. Phylogenetic analysis revealed distinct evolutionary paths for these genes, with *Ipper2l* forming a separate clade from *Ipper2*. Tissue-specific expression analysis showed differential expression of period genes across tissues, with *Ipper1b* exhibiting the highest expression in the intestine and *Ipper2* being predominantly expressed in the liver. Statistical analysis confirmed significant differences in the expression levels between tissues (*p* < 0.05), supporting the tissue-specific roles of these genes. Notably, under strict feeding schedules, we observed significant modulation of rhythmic expression in both the brain and liver, with a notable shift in the peak expression times and amplitude changes aligned with the feeding time. These results suggest that feeding serves as a critical Zeitgeber, entraining circadian rhythms in key tissues and potentially enhancing metabolic efficiency. These results demonstrated that feeding schedules play a key role in modulating circadian gene expression in channel catfish. This study provides insights into the evolutionary divergence and functional roles of the period gene family in channel catfish, showing how feeding schedules modulate circadian gene expression in the brain and liver. These findings have potential applications in optimizing feeding strategies for improving fish health and growth in aquaculture.

## 1. Introduction

Organisms adapt to environmental changes through circadian rhythms, also known as biological clocks, which regulate daily cycles in response to factors such as light and temperature. These rhythms are vital for maintaining various physiological functions, and understanding their molecular regulation is key to improving aquaculture practices [1,2]. The circadian system comprises core oscillators that integrate input and output from external and internal signals through various channels [3]. The molecular mechanisms that underlie the circadian system are based on transcriptional/translational auto-regulatory feedback loops involving a set of clock genes.

In vertebrates, three transcription factors (CLOCK, NPAS2, and BMAL) function as positive regulators [4]. In conjunction with BMAL1, NPAS2 or CLOCK form dimers that bind to specific elements (e.g., E-box and 5′-CANNTG-3′) in the promoter regions of downstream clock-controlled genes to regulate their transcription [5]. These clock genes are typically regulated negatively. The accumulated negative regulatory protein dimers then bind to the positive regulatory dimers on DNA, inhibiting transcriptional activity and creating a negative feedback loop [2]. Once inside the nucleus, these dimers suppress the positive regulatory factors, thereby modulating their own expression. Additionally, subsequent phosphorylation and ubiquitination of the negatively regulated proteins result in their degradation, which reactivates the positive regulatory dimers.

Period family genes are integral to the negative feedback loops of the circadian clock mechanism and are highly conserved across different species. Period and cryptochrome proteins form a heterodimer that binds to positive regulatory protein dimers, such as Clock-Bmal1, which interact with the E-box in the promoters and enhancers of numerous genes. The first identified clock gene, *period* (*per*), was discovered through studies of mutations that altered the free-running circadian rhythm of motor activity in *Drosophila* [6]. In vertebrates, the period family has three members, and mammals have three period homologs (period1–3). They play roles in circadian clock regulation, reproduction, development, and immunity [7,8]. Teleost fish, the most diverse group of vertebrates, exhibit tremendous diversity in morphology and behavior to adapt to different environments, leading to differences in the number and function of period family genes across species. For instance, zebrafish (*Danio rerio*) possess two *per1* (*per1a* and *per1b*), one *per2*, and one *per3* gene, while medaka (*Oryzias latipes*) and pufferfish (*Fugu* and *Tetraodon*) each have two *per2* (*per2a* and *per2b*), one *per1*, and one *per3* gene. Sticklebacks (*Gasterosteus*) have *per2a*, *per2b*, and one *per1* gene but lack *per3* [9]. The period family in fish originated from a single ancestral period gene, followed by two or three rounds of genome duplication that resulted in functional diversification [8].

Period family genes regulate various physiological, behavioral, and reproductive rhythms in both vertebrates and invertebrates. In mammals, the expression of circadian clock genes oscillates with circadian rhythms in the brain and peripheral organs and is driven by the suprachiasmatic nucleus (SCN), an oscillatory pacemaker located in the hypothalamus. In mice, constitutive expression of *per2* in the SCN disrupts circadian rhythms in fibroblasts and liver and can even lead to the loss of behavioral circadian rhythms [10].

Instead of the SCN, the pineal gland of non-mammalian vertebrates serves as a light-sensitive organ, as it contains an intrinsic circadian oscillator that influences daily rhythms and seasonal changes [11]. In zebrafish, the light-sensitive protein PER1b exhibits visual function, and its mutation affects visual behavior [12]. The liver also maintains its circadian rhythm to maximize energy conservation and protect cells from damage. Period genes are crucial for sustaining this rhythm and are essential for the liver’s metabolic homeostasis [7]. In Senegalese sole (*Solea senegalensis*), oscillations of *per1* and *per2* have been observed [12].

The immune system’s physiology follows a 24 h circadian pattern, with the majority of immune cells exhibiting circadian gene activity that affects cellular functions, cytolytic activities, and cytokine production. Disorders of circadian rhythms and immune system dysregulation, along with cytokine expression, are significantly influenced by polymorphism of PER3 in humans [13,14]. A previous study linked the PER3 polymorphism to persistent inflammation markers, including interleukin 1, interleukin 6, interferon-gamma, and tumor necrosis factor-alpha [8].

Many researchers have explored the relationship between period family genes and circadian rhythm, including daily rhythms and seasonal rhythms, as well as visual function and immune disorders in species such as humans, mice, fruit flies, and various fish [15,16]. Recent studies have demonstrated that mutation in period family genes can lead to alterations in metabolism, growth, and reproduction in zebrafish [17]. Moreover, *per2* is associated with the entrainment of both central (brain) and peripheral (liver) tissues by light and feeding cycles in goldfish (*Carassius auratus*). To date, however, no studies have characterized the regulatory mechanisms of period genes in channel catfish, which is a nocturnal fish that emerges at dusk to feed.

The channel catfish is now extensively cultured across various provinces in China. Its feeding rhythm is particularly crucial in aquaculture, as it influences the growth characteristics of the fish. Despite extensive research on the channel catfish in areas such as growth, immunity, and reproduction, little is known about its circadian clock gene. Therefore, the goal of this study was to analyze the circadian system and entrainment pathways in channel catfish using cloning, tissue distribution, phylogenetic relationships, and synteny analysis. The identification and characterization of the period gene family in channel catfish will contribute valuable insights that are applicable to aquaculture practices.

## 2. Materials and Methods

### 2.1. Experimental Animals and Sampling

Healthy channel catfish juveniles (100–150 g) were collected from the National Genetics and Breeding Center of Channel Catfish (Nanjing, Jiangsu Province, China) in June 2023. The fish were maintained in aquaria with a recirculating freshwater system under a constant dark environment. The light cycle was set to 12 h of light (8:00–20:00) and 12 h of darkness (20:00–8:00 the following day), and this light cycle remained consistent throughout the study. After a 3-week acclimatization period, the fish were randomly divided into two groups: the random feeding group (RF, control) and the strict feeding group (SF), with 36 fish in each group. Each group was further subdivided into three replicate tanks (*n* = 12 fish per tank) to ensure experimental reliability and reproducibility.

In the experiment, fish in the SF group were fed at 14:00 each day, while fish in the RF group were fed at random times. After 3 weeks, fish were euthanized using MS-222 for anesthesia before tissue collection. The following tissues were collected from each fish: brain, head kidney, kidney, muscle, spleen, blood, testis, ovary, liver, and intestines. Total RNA was extracted from these tissues for subsequent gene cloning and gene expression profiling studies.

### 2.2. Molecular Cloning and Tissue Distribution of Expression of Period Family Genes

Total RNA was extracted from the livers of channel catfish using the AllPrep RNA Mini Kit (Qiagen, Hilden, Germany) in accordance with the manufacturer’s guidelines. Subsequently, cDNA was synthesized from the RNA using a cDNA synthesis mix kit (Takara, Dalian, China). To specifically amplify the full-length coding regions of the channel catfish period family genes, primers were designed based on the predicted mRNA sequences (XM_053681396.1, XM_053688566.1, XM_053673630.1, and XM_017450144.3) from NCBI. The sequences of these primers are provided in Table S1. A 20 μL PCR reaction mixture, which included 10 pM (1.2 μL) primers, 100 ng (1.9 μL) cDNA, 10 μL of mix from Vazyme (Vazyme, Nanjing, China), and 6.9 μL of H_2_O, was used to amplify the period family genes. The essential PCR settings were as follows: initial incubation at 95 °C for 10 min; 30 cycles at 95 °C for 15 s and 55 °C for 30 s; and final extension at 72 °C for 1 min. PCR products were isolated from agarose gels using the Universal DNA Purification Kit (Takara, Dalian, China). The purified amplification products were then ligated into the pMD19 vector using the pMD™ 19-T Vector Cloning Kit (Takara, Dalian, China). These constructs were subsequently transformed into competent *Escherichia coli* DH5α cells (Takara, Dalian, China). After culturing overnight, colony PCR was performed to select positive clones. Positive bacterial solution was sent to Shanghai Shenggong Biological Company (Shanghai, China) for sequencing.

To assess the tissue-specific expression of the period family genes, quantitative real-time PCR (qRT-PCR) was conducted. Total RNA was extracted from the tissues collected from the channel catfish, as described above, and subsequently converted into cDNA following previously established procedures. Primer5 software was employed to design four pairs of primers for the qRT-PCR (Table S2). The real-time PCR assays were then executed on an Eppendorf Realplex Real-Time PCR system (Eppendorf, Hamburg, Germany) using TB Green Fast qPCR Mix (Takara, Dalian, China) following the instructions provided by the manufacturer. Alpha-tubulin served as the housekeeping reference gene. The process for real-time quantification involved initial denaturation at 95 °C for 15 s, followed by annealing and extension at 55 °C for another 15 s. The fluorescence intensity of TB Green dye was measured at 55 °C after the extension phase of each cycle was complete. The 2^−△△Ct^ method was used to calculate the relative expression for each examined tissue, and melting curves were used to verify the specificity of primers for each period family gene.

### 2.3. Bioinformatic Analysis

Using Mega X software, we conducted an amino acid sequence alignment of the period gene family proteins from closely related species, including *Danio rerio* (*D. rerio*), *Silurus asotus* (*S. asotus*), *Silurus meridionalis* (*S. meridionalis*), *Siniperca chuati* (*S. chuati*), *Micropterus salmoides* (*M. salmoides*), and *Ictalurus punctatus* (*I. punctatus*). This alignment was performed to assess the sequence homology and evolutionary relationships among these species. The phylogenetic tree of period family proteins was constructed using protein sequences from 20 different species obtained from the NCBI database (https://www.ncbi.nlm.nih.gov/protein, accessed on 12 June 2023). The amino acid sequences of period proteins of those species were aligned using Mega X software (version 7.0; MEGA software, Tempe, AZ, USA). Subsequently, the aligned amino acid datasets were used to generate a phylogenetic tree in MEGA X through the neighbor-joining method with 1000 bootstrap replicates. The optimal model for protein evolution was determined, and the Jones–Taylor–Thornton + Nearest-Neighbor Interchange algorithm distances were selected to automatically create initial tree models (Standard-NJ/BioNJ) for evaluation. To investigate syntenic relationships, we compared target genes and adjacent gene orthologs from channel catfish (*I. punctatus*) with those in other teleosts (including *D. rerio*, *M. salmoides*, *Oncorhynchus mykiss* (*O. mykiss*), *Salmo salar* (*S. salar*), *S. meridionalis*, *Siniperca chuatsi*, *Labeo rohita* (*L. rohita*), *Pangasianodon hypophthalmus* (*P. hypophthalmus*), and *Tachysurus fulvidraco* (*T. fulvidraco*)). This comparison began at the chromosome scale using the Synteny Database and then focused on the local gene neighborhood using the Genomicus Browser to obtain detailed ortholog information across individual chromosomes. The protein–protein interaction network of channel catfish period proteins was established using the STRING database (https://string-db.org/). Sequence logos of the conserved domains were produced with the WebLogo 3 program (https://weblogo.threeplusone.com).

### 2.4. In Silico Analyses

Models of the period proteins were generated through comparative modeling using the SWISS-MODEL web server (https://swissmodel.expasy.org/) and subjected to energy minimization for structural optimization [18]. Differences in the secondary structures and 3D models among the channel catfish period proteins were identified using the Swiss PDB Viewer 3.7 program. The predicated 3D structure of the conserved domain of period proteins was also analyzed using SWISS-MODEL. NetPhos-3.1 (https://services.healthtech.dtu.dk was used to predict the putative C-terminal phosphorylation sites).

## 3. Results

### 3.1. Molecular Cloning and Tissue Distribution of the Channel Catfish Period Family Genes

Four period family genes were identified in channel catfish: *Ipper1b*, *Ipper2*, *Ipper2l*, and *Ipper3*. These genes encode proteins with coding sequences of 4332, 4122, 3942, and 3837 bp that correspond to 1443, 1373, 1313, and 1279 amino acids, respectively (Figures S1–S4). Table 1 lists the molecular weights, isoelectric points (pI), GRAVY values, amino acid counts, and compositions of various amino acid types for the four period proteins of channel catfish (IpPER1b, IpPER2, IpPER2l, and IpPER3). PER1b had the highest molecular weight at 154.56 kDa, followed by IpPER2l (149.31 kDa), IpPER2 (143.85 kDa), and IpPER3 (138.48 kDa). The pIs range was narrow; IpPER1b had the lowest pI (5.81), and IpPER2 had the highest pI (5.98). All proteins had negative GRAVY values, indicating hydrophilic characteristics; IpPER2 was the most hydrophilic (−0.699), and IpPER1b was the least hydrophilic (−0.573).

Our analysis of amino acid type classes revealed that aliphatic residues were most and least abundant in IpPER3 (30.36%) and IpPER2 (27.04%), respectively. Aromatic residues were fairly consistent, peaking in IpPER2 (6.40%). Basic residues ranged from 11.16% in IpPER1b to 12.82% in IpPER3, while acidic residues varied more widely, from 19.33% in IpPER1b to 22.00% in IpPER2l. Additionally, the proportions of amino acids related to tRNA synthetase classes showed that class I enzymes ranged between 40.06% (IpPER1b) and 41.20% (IpPER2), and class II enzymes varied from 58.80% (IpPER2) to 59.94% (IpPER1b and IpPER2l). These results highlight significant differences in molecular characteristics and amino acid compositions among the period proteins in channel catfish, suggesting potential variations in their functional roles and adaptations in circadian rhythm regulation across different teleost species.

**Figure 1:**
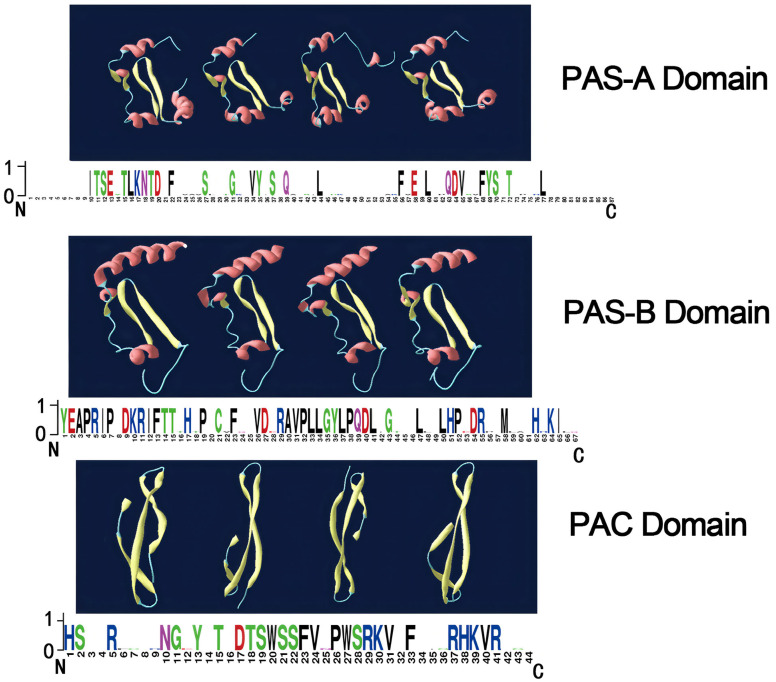
Structural representations of conserved domains in IpPER proteins, showcasing the three-dimensional architecture of PAS-A, PAS-B, and PAC domains. Structural alignments and weblogo plots depict domain conservation across the IpPER protein family, underscoring the evolutionary conservation and functional relevance of these motifs in regulatory mechanisms. This figure highlights the domain architecture conservation among IpPER proteins, emphasizing the evolutionary stability of key functional sites across species.

The channel catfish period proteins also have highly conserved domains, including PAS-A, PAS-B, and PAC domains (Figure 1). These domains mediate the interactions between period proteins and the BMAL1-CLOCK heterodimer. The conserved domain sequences were aligned with the Mega X program and WebLogo server (http://weblogo.berkeley.edu/logo.cgi) (Figure 1). The 3D models of the conserved domains presented in Figure 1 show that the PAS-A domains contain four α-helices and four β-sheets, while the PAS-B domains consist of three α-helices and three β-sheets. The PAC domains, meanwhile, are composed of four β-sheets.

### 3.2. Phylogenetic Analysis and Multiple Alignment

**Figure 2:**
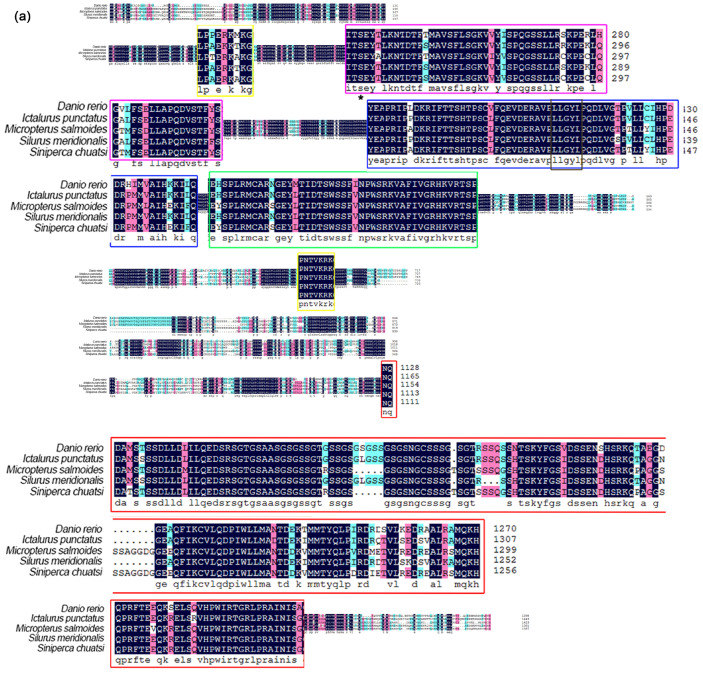

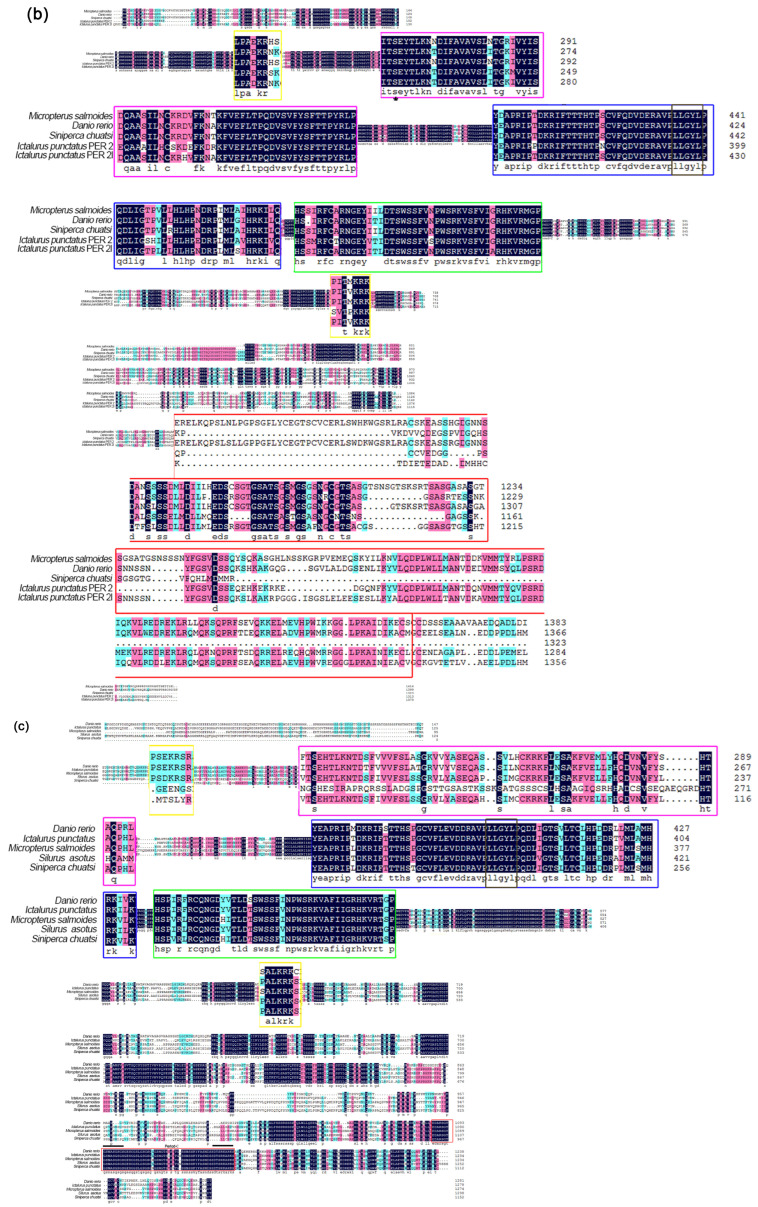
Comparative analysis of IpPER proteins and structural domain conservation across teleosts. (**a**–**c**) Sequence alignments of IpPER1b, IpPER2, IpPER2l, and IpPER3 proteins from *I. punctatus* with homologous period proteins from other teleost species. Conserved regions are highlighted with color-coded boxes: PAS-A domain (purple), PAS-B domain (blue), PAC domain (green), nuclear localization signal (NLS, yellow), and C-terminal conserved domain (red). Conserved phosphorylation sites are indicated by asterisks (*), marking residues critical for post-translational regulation.

In the phylogenetic analysis of channel catfish, the *Ipper1b* gene had the highest similarity with *S. meridionalis* species (84.89%), whereas its similarity with other species ranged from 65.85% to 74.21% (Figure 2a). Similarly, the *Ipper2* gene displayed significant similarities with other fish: 75.3% with *D. rerio*, 60.61% with *M. salmoides*, and 73.04% with *S. chuatsi*. In contrast, the *Ipper2l* gene exhibited considerably lower similarity with the same species, with values of 56.4%, 48.8%, and 46.27%, respectively, compared to *Ipper2* (Figure 2b). As a result, *Ipper2* clustered with these species in the phylogenetic tree, while *Ipper2l* formed a distinct branch, reflecting its divergent evolutionary trajectory. For the *Ipper3* gene, the similarity with *D. anio*, *M. salmoides*, *S. asotus*, and *S. chuatsi* ranged between 65.45% and 55.19% (Figure 2c). These findings collectively suggest distinct evolutionary pathways for the period gene family in *I. punctatus* and related species. The amino acid analysis showed that two highly conserved NLS are present in the period proteins of channel catfish and other fish species (Figure 2a–c).

**Figure 3:**
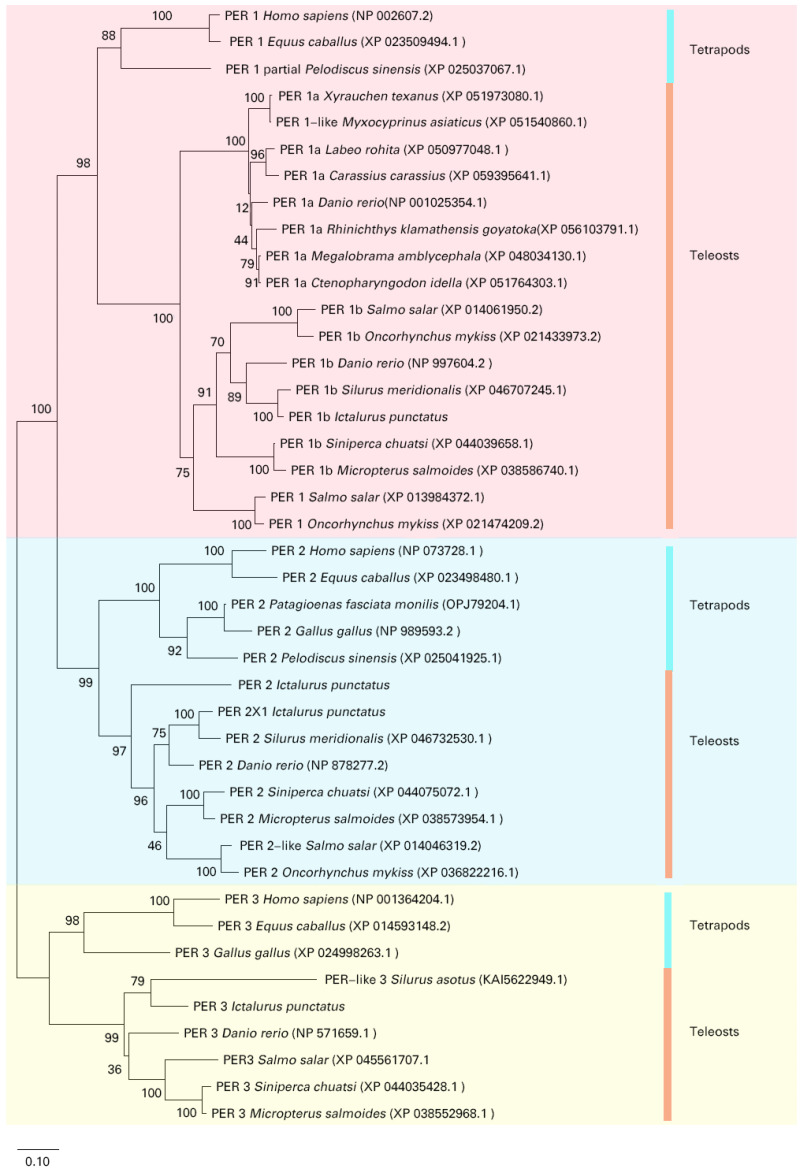
Phylogenetic tree of period proteins. The tree is divided into three main branches: PER1, PER2, and PER3. Within the PER1 branch, the period protein from *I. punctatus* (IpPER1b) clusters with those from *S. meridionalis*, indicating a close evolutionary relationship. The PER2 branch includes IpPER2, which forms a distinct lineage alongside zebrafish and other PER2 proteins. In the PER3 branch, the period protein from *I. punctatus* (IpPER3) groups with those from *Silurus asotus*, while a separate cluster consists of PER3 proteins from *Homo sapiens*, *Equus caballus*, and *Gallus gallus*. This tree illustrates the evolutionary divergence and conservation of period proteins across vertebrate species, underscoring their functional significance.

Multiple alignment of period genes across 20 fish species revealed that they are cleanly classified into groups: *per1*, *per2*, and *per3* (Figure 3). Proteins of the same subfamily are expected to cluster within the same branch. In the phylogenetic tree, the branch for *per2* includes both channel catfish *per2* and *per2l*. Channel catfish *per1* clusters with *per1b* from other species, leading to its designation as *Ipper1b*.

**Figure 4:**
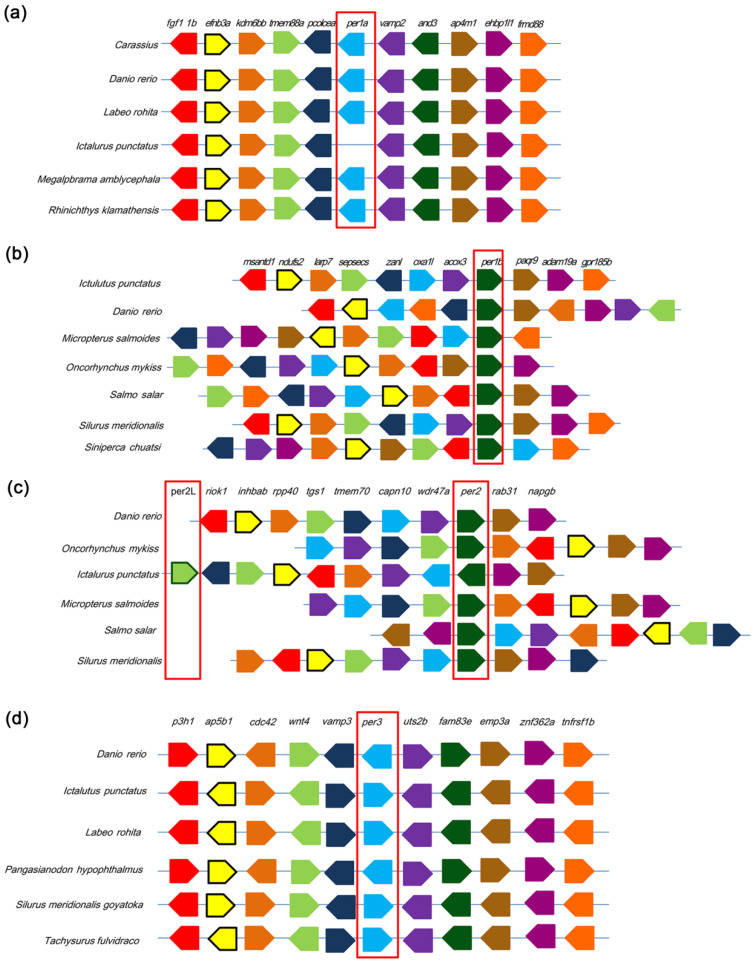
Phylogenetic alignment of the period protein family in *I. punctatus*. (**a**) The alignment for the *per1a*, showing its presence in 6 related species while being absent in *I. punctatus*. (**b**) The *per1b* gene, which is conserved across all 7 analyzed fish species. (**c**) This focuses on *per2*, found in 6 species, with *per2l* uniquely identified in *I. punctatus*. (**d**) The alignment for *per3*, present in 6 species. This analysis emphasizes the evolutionary relationships and conservation patterns of the period gene family in *I. punctatus*. Red boxes: period proteins.

Comparative synteny analysis of the circadian clock loci *per1a*, *per1b*, *per2*, and *per3* across multiple teleost genomes revealed both conserved and lineage-specific structural variations. At the *per1a* locus, the conserved adjacency and orientation of *pcsk9* and *vamp2* flanking the *per1a* block in five reference species is disrupted in channel catfish (*I. punctatus*), where *per1a* is absent, and *pcsk9* and *vamp*2 are juxtaposed within an otherwise intact gene cluster (Figure 4a). For *per1b*, despite interspecies rearrangements of distal neighbors, *per1b* consistently resides immediately upstream of *paqr9* in six teleosts, with only *S. chuatsi* diverging (Figure 4b). The *tmem70*–*per2*–*capn10* module exhibits a uniform transcriptional orientation in all surveyed genomes except *M. salmoides*; however, flanking regions of *per2* display high variability, and *I. punctatus* uniquely harbors a tandem-duplicated *per2l* with no orthologous context in other species (Figure 4c). Finally, the *per3* locus shows an invariant ten-gene order (*p3h1* through *tnfrsf1b*) and conserved orientation across six teleosts, underscoring strong evolutionary constraint (Figure 4d). Together, these findings delineate a lineage-specific deletion of *per1a* in channel catfish, reveal the uniform conservation of the *tmem70*–*per2*–*capn10* module amidst pronounced variability in *per2* flanking regions and the species-specific tandem duplication of *per2L* in *I. punctatus*, and demonstrate pervasive stability of core syntenic frameworks alongside dynamic remodeling and paralog expansion in teleost circadian gene clusters.

### 3.3. Protein–Protein Interaction Network Analysis

**Figure 5:**
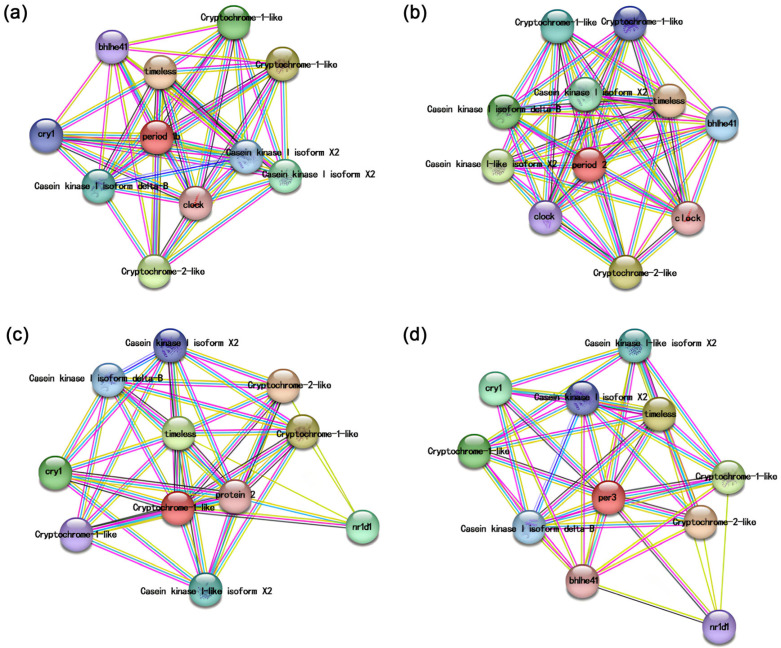
Protein–protein interaction network of period family members in *I. punctatus.* This network illustrates the interactions among various period proteins, highlighting potential functional relationships and pathways. Each node represents a period protein, while edges indicate direct interactions. Subfigures (**a**–**d**) represent the interaction networks of IpPER1, IpPER2, IpPER2L, and IpPER3, respectively.

Protein–protein interaction network analysis elucidates the interactions among genes. Utilizing the STRING database, we predicted proteins that interact with period family genes. The results indicated that the proteins significantly interacting with the channel catfish circadian genes include CRY and TIMELESS, also interacting with casein kinase. Notably, IpPER1b and IpPER2 interact with CLOCK, and IpPER1b, IpPER2, and IpPER3 interact with BHLHE41. Furthermore, only IpPER2l interacts with NR1D1 (Figure 5).

### 3.4. Tissue Distribution of I. punctatus Period Genes

**Figure 6:**
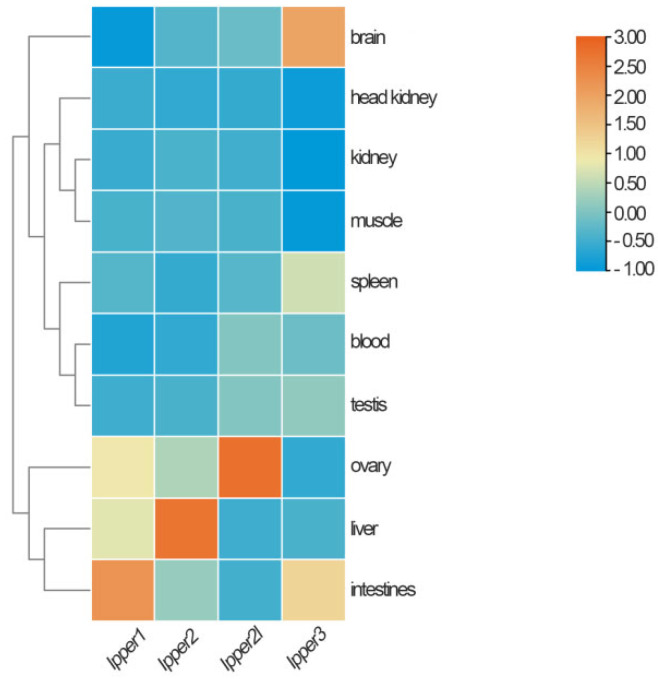
Expression levels of period genes across various tissues. The data present individual period gene expression values (*Ipper1*, *Ipper2*, *Ipper2l*, and *Ipper3*) in different tissues: brain, liver, kidney, spleen, muscle, intestine, head kidney, blood, testis, and ovary. Data are represented as means with standard deviations from multiple biological replicates.

To understand the function of each period gene in channel catfish, qRT-PCR was applied to measure their expression in different tissues. Transcripts for the four period genes were detected in all 10 tested tissues, but their expression levels differed among tissues. *Ipper1b* was predominantly expressed in the intestine, whereas *Ipper2* was highly expressed in the liver. *Ipper2l* was primarily expressed in the ovary, and *Ipper3* was most highly expressed in the brain, followed by the intestines. However, the expression of all four genes was low in the head kidney, kidney, and muscle. These findings are illustrated in Figure 6.

### 3.5. Diurnal Expression of Period Family Genes in the Brain and Liver

The expression patterns of the period family genes were examined using qRT-PCR for the analysis of brain and liver samples from fish cultured under the RF and SF feeding regimes. Rhythmic expression of these genes was observed in both the brain and liver under both conditions, but strict feeding notably affected their expression profiles.

**Figure 7:**
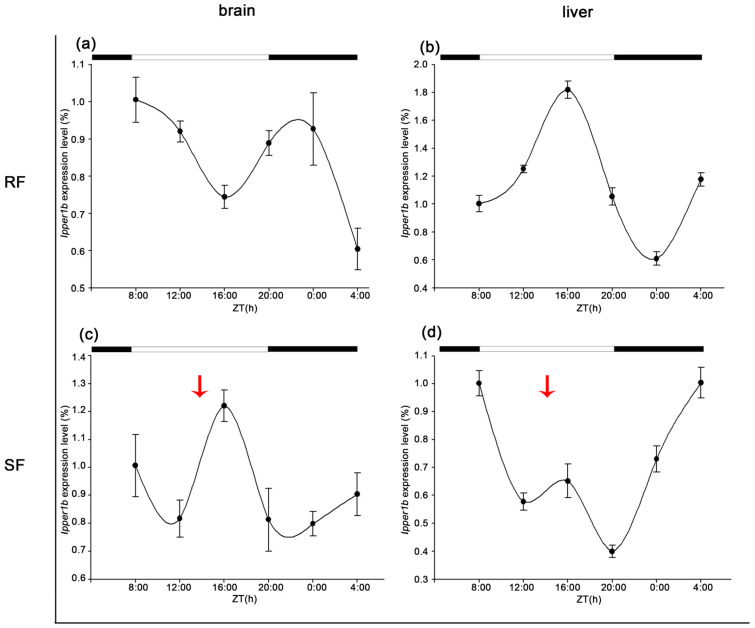
Expression patterns of IpPER1b in brain and liver under different feeding regimes. (**a**) Expression of IpPER1b in the brain under random feeding (RF) over 24 h. (**b**) Expression of IpPER in the liver under RF. (**c**) Expression of IpPER1b in the brain under strict feeding (SF). (**d**) Expression of IpPER1b in the liver under SF. qRT-PCR analysis shows rhythmic expression of IpPER1b in both tissues under RF and SF conditions. In the brain, peak levels occurred at 8:00 and 23:00 for the RF group. For the SF group, peak expression shifted to around 14:00. In the liver, light exposure typically enhances expression, but under SF, the peak normally observed at 16:00 is suppressed, indicating significant effects of the feeding regimes on gene expression profiles. The red arrows indicate the feeding time for SF.

In the brain, the peak expression of *Ipper1b* occurred at 8:00 and 23:00 in the RF group, with relative expression levels of 1.00 and 0.95, respectively. Following 3 weeks of strict feeding, the expression phase of *Ipper1b* shifted to approximately 8 h later, resulting in expression amplitudes of 0.4 and 0.6 at 14:00. In the liver, the expression pattern of *Ipper1b* was also modified; light exposure typically enhanced its expression, but under strict feeding, light exposure decreased its expression, thereby suppressing the peak that normally occurred at 16:00 (Figure 7).

**Figure 8:**
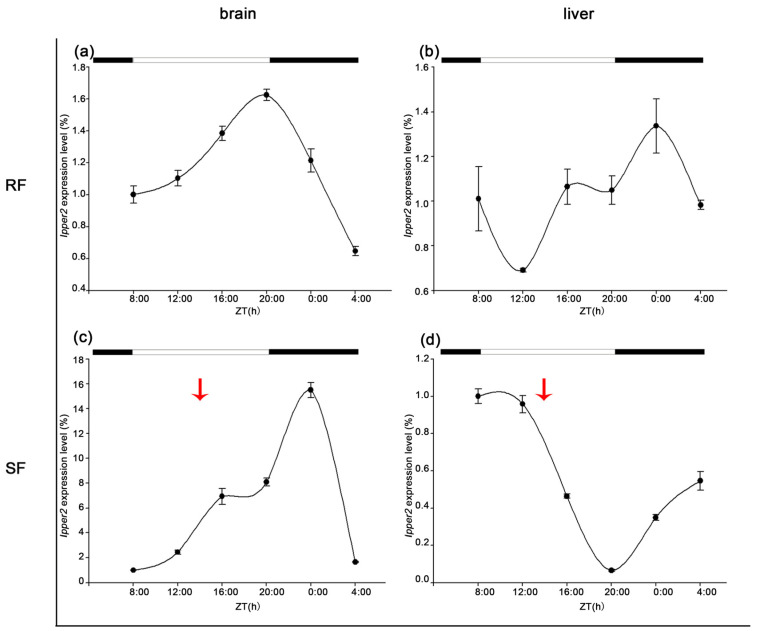
Expression trends of IpPER2 in the brain and liver under different feeding regimes. (**a**) The 24 h expression profile of IpPER2 in the brain under the RF (random feeding) condition shows a peak amplitude of 1.625 at 20:00. (**b**) The liver expression of IpPER2 under RF reveals a maximum amplitude of 15.51 at 24:00, indicating a significant increase throughout the cycle. (**c**) In the brain under SF (strict feeding), IpPER2 expression peaks at 1.385 at 16:00, demonstrating a delay of 4 h compared to RF. (**d**) The liver expression under SF has a lower peak of 0.348 at 24:00, with a prolonged expression cycle of 28 h, despite stable amplitude levels. Notably, the comparison of RF and SF indicates that strict feeding alters the timing and amplitude of expression, particularly in the brain, where the amplitude increases from 1.6 (RF) to 1.385 (SF), and in the liver, where RF yields significantly higher values. The red arrows indicate the feeding time for SF.

The expression of *Ipper2* differed significantly between the RF and SF groups. In the brain, strict feeding delayed the peak expression by 4 h and increased the amplitudes from 1.6 to 16. In the liver, strict feeding prolonged the expression cycle from 24 to 28 h, although the amplitudes remained relatively stable (Figure 8).

**Figure 9:**
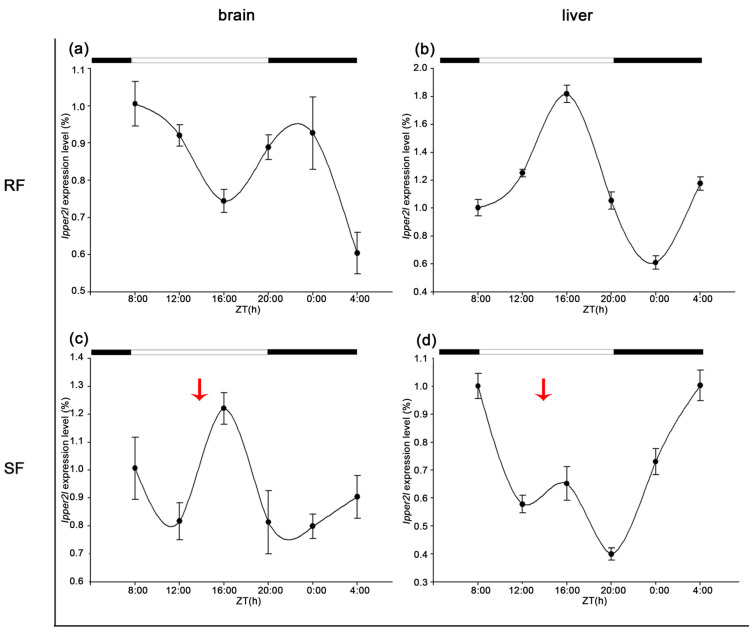
Diurnal expression patterns of IpPER2L in brain and liver under different feeding conditions. (**a**) The 24 h expression trend of IpPER2L in the brain under random feeding (RF) conditions, peaking at 24:00 with a value of 4.59. (**b**) RF expression in the liver, with a maximum at 24:00 (36.57), indicating a stronger amplitude compared to brain expression. (**c**) The expression trend in the brain under restricted feeding (SF) conditions, where the peak at 20:00 (2.98) reflects an 8 h delay compared to RF conditions. (**d**) Liver expression under SF, where the peak is observed at 12:00 (3.81), demonstrating a reduction in the cycle length to 24 h, thus synchronizing liver expression with brain expression. These data suggest that the feeding regimen significantly alters the circadian regulation of IpPER2L, with SF conditions leading to a more synchronized expression pattern between the two tissues. The red arrows indicate the feeding time for SF.

*Ipper2l* displayed periodic expression in both the brain and liver. In the brain, the expression cycle was delayed by 8 h in the SF group. Conversely, in the liver, strict feeding shortened the cycle length from 48 to 24 h, synchronizing liver expression with that in the brain (Figure 9).

**Figure 10:**
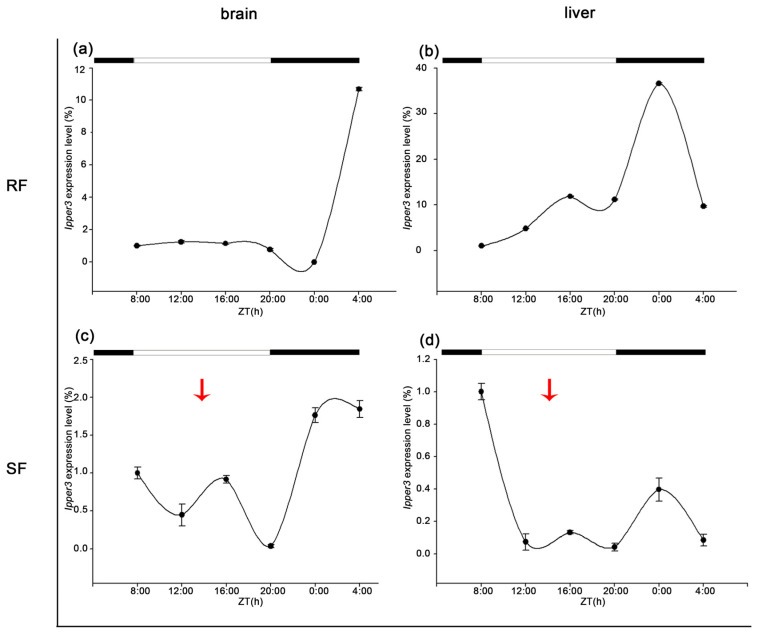
Expression patterns of IpPER3 in brain and liver under different feeding conditions. (**a**) The 24 h expression trend of IpPER3 in the brain under random feeding (RF) conditions, showing no rhythmic variation, with a peak of 10.683 at 24:00. (**b**) The expression trend in the liver under RF conditions displays rhythmic expression, peaking at 36.604 at 24:00. (**c**) The 24 h expression trend of IpPER3 in the brain under strict feeding (SF) conditions, with a peak of 1.847 at 0:00. (**d**) In the liver under SF conditions, IpPER3 exhibits rhythmic expression with a peak of 0.397 at 24:00, significantly delayed by 8 h compared to RF conditions. These results indicate a pronounced alteration in expression dynamics due to the feeding schedule, highlighting the influence of feeding conditions on IpPER3 regulation. The red arrows indicate the feeding time for SF.

*Ipper3* was not expressed rhythmically in the RF group, but it displayed rhythmic expression in the liver, with a peak at midnight. Under strict feeding conditions with feeding scheduled at 14:00, the expression pattern was altered. *Ipper3* was rhythmically expressed in both the brain and liver, with peak expression in the liver delayed by 8 h (Figure 10).

## 4. Discussion

### 4.1. Evolutionary Divergence and Functional Specialization of Channel Catfish Period Genes

A hallmark of the vertebrate circadian clock system is the presence of multiple copies of core clock genes, a feature reflecting both evolutionary history and species-specific adaptations [19,20]. In Drosophila, the single *dper* gene corresponds to three orthologous period genes in mammals: *per1*, *per2*, and *per3* [21]. Each mammalian period gene exhibits distinct but overlapping roles in maintaining circadian rhythms [2].

In teleost fish, the evolutionary landscape of the period gene family has been further shaped by an additional whole-genome duplication (WGD) event, resulting in an expanded and diversified repertoire compared to mammals [22]. For example, zebrafish possess four period genes: *per1a*, *per1b*, *per2*, and *per3* [23]. However, this gene expansion has not been uniform across teleosts. Some species, like pufferfish (*Fugu* and *Tetraodon*), have lost *per3*, retaining only *per1* and *per2* [24]. Such gene losses are likely the result of relaxed selection and functional redundancy following duplication [25].

These evolutionary modifications are thought to facilitate species-specific tuning of circadian rhythms to local environmental and ecological contexts [26]. In zebrafish, the retention of both *per1a* and *per1b*, along with *per3*, may enhance flexibility in responding to diel variations in light and temperature [27]. Conversely, in species like goldfish (*C. auratus*) and channel catfish, gene repertoires have diverged further. For instance, goldfish retain *per1a* and employ *Per2* for cold-acclimated gluconeogenic rhythms [28]. Channel catfish, which lack *per1a*, may compensate via functional expansion of *Ipper1b*, an adaptation likely linked to their benthic, low-light habitats.

Similarly, reproductive timing mechanisms appear to differ between species. Nile tilapia (*Oreochromis niloticus*) utilizes *per1b* to coordinate feeding and reproductive cycles [29], while in channel catfish, the gonad-specific expression of *Ipper2l* may decouple reproduction from photoperiod constraints, supporting opportunistic spawning, an advantage for continuous breeders in fluctuating environments.

In this study, four period genes were identified in channel catfish: *Ipper1*, *Ipper2*, *Ipper2l*, and *Ipper3*. The absence of *per1a*, despite conserved syntenic flanking genes, suggests a lineage-specific gene loss consistent with patterns observed across teleosts [30]. The retention of two *per2*-like genes (*Ipper2* and *Ipper2l*) further highlights the evolutionary diversity and likely functional specialization of this gene family in channel catfish, shaped by ecological demands such as light availability and reproductive flexibility [31].

Collectively, these findings underscore the remarkable evolutionary plasticity of the period gene family within teleost, shaped by genome duplication, gene retention, and selective loss. The varying repertoires of period genes among species such as zebrafish, pufferfish, goldfish, and channel catfish reflect adaptive responses to ecological and physiological demands, including differences in photic environments, reproductive strategies, and energy regulation. The loss of per1a in channel catfish, alongside potential functional expansion of *Ipper1b* and the retention of both *Ipper2* and *Ipper2l*, exemplifies how gene loss may be compensated through regulatory specialization. Moreover, the integration of circadian genes with metabolic and reproductive processes highlights their broader functional relevance beyond timekeeping. As such, teleost fish represent a valuable model for elucidating the evolution, divergence, and specialization of circadian regulatory networks across vertebrates.

### 4.2. Period Family Gene Function Is Related to Energy Metabolism and Growth

Our results demonstrated that both random feeding and strict feeding conditions influence the rhythmic expression of period genes in the brain and liver of the channel catfish. However, strict feeding appears to have a more pronounced effect on the expression profiles of these genes, suggesting that a fixed feeding schedule exerts a stronger influence on the synchronization of these biological rhythms. This observation aligns with earlier studies of other fish species in which the feeding time functioned as a Zeitgeber, influencing the phase and amplitude of clock gene expression in peripheral tissues such as the liver [32,33]. In particular, fixed feeding times have been shown to enhance the entrainment of circadian rhythms in various tissues, thereby impacting physiological processes such as metabolism and digestion [34]. Furthermore, research on zebrafish and goldfish has demonstrated similar outcomes, where consistent feeding schedules led to a more robust expression of clock genes in peripheral organs, indicating a conserved mechanism among teleost fish [35,36].

Under strict feeding conditions, the peak expression of *Ipper1b* in the brain shifted by approximately 8 h, with a reduction in expression amplitude. This shift indicates that the feeding schedule may override the endogenous rhythm, aligning the peak expression with feeding time rather than light–dark cycles [37,38]. Notably, this 8 h phase delay exceeds the 6 h shift observed in Nile tilapia hepatic *Per1b* under restricted feeding [39], potentially reflecting stronger selective pressure for catfish to synchronize metabolism with unpredictable benthic food availability. Conversely, goldfish prioritize PER2-driven gluconeogenic precision under stable cold conditions [28], underscoring how ecological niche shapes circadian adaptability. Such a phase delay suggests that the brain’s circadian system adapts to feeding time, potentially prioritizing energy intake and processing over the environmental light–dark cycle [40]. Additionally, the substantial increase in amplitude (from 1.6 to 16) observed for *Ipper2* under strict feeding suggests that the brain’s response to feeding time is highly sensitive. This might be a mechanism to optimize metabolic processes in response to a predictable feeding schedule [40,41].

The liver’s role as a metabolic organ makes it highly responsive to changes in feeding patterns [40,42]. In this study, the rhythmic expression of *Ipper1b* in the liver was modulated by light exposure under random feeding conditions, while strict feeding altered this response, reducing expression during light exposure and suppressing the usual peak. This suggests that under strict feeding conditions, the liver’s response becomes more tightly coupled to feeding time rather than light cues [16]. The lengthening of the expression cycle of *Ipper2* from 24 to 28 h in the liver under strict feeding further implies a desynchronization with the natural light–dark cycle, potentially reflecting an adaptation to optimize nutrient processing in response to the fixed feeding schedule [43].

The synchronization of *Ipper2l* expression between the brain and liver under strict feeding conditions was observed, and the liver cycle was shortened from 48 to 24 h. This result indicates that feeding can serve as a unifying cue to synchronize rhythms across different tissues [44]. This synchronization may improve the efficiency of metabolic processes, ensuring that both the central and peripheral clocks work in harmony to manage energy use and storage effectively [45].

Interestingly, *Ipper3* was not rhythmically expressed in the liver under random feeding conditions but displayed a clear rhythmic pattern in both the brain and liver under strict feeding, with a peak at midnight. This feeding-induced rhythmicity mirrors zebrafish *per3* light responsiveness [46], suggesting evolutionary repurposing of *Ipper3* to transduce nutrient signals rather than photic cues, a plausible adaptation for nocturnal benthic feeders. This suggests that strict feeding can induce rhythmicity in genes that are otherwise non-rhythmic, highlighting the importance of feeding schedules as a modulator of gene expression patterns [47]. The 8 h delay in the liver’s peak expression of *Ipper3* under strict feeding aligns with the feeding time, which supports the idea that this gene’s rhythmicity is closely tied to the timing of nutrient availability [48]. Such findings emphasize the role of feeding as a Zeitgeber that can influence the synchronization of peripheral oscillators with nutrient intake.

The adaptation of period gene expression patterns in response to feeding schedules may be linked to the ecological habits of channel catfish, which are opportunistic feeders and often display nocturnal feeding behaviors. In natural environments, the availability of food is not always predictable, and fish must adapt their feeding and metabolic rhythms accordingly [49]. Our results suggest that strict feeding schedules serve as a potent Zeitgeber, modulating the circadian rhythms in channel catfish. This phenomenon aligns with findings in other species, such as zebrafish, where feeding times have been shown to influence clock gene expression in peripheral tissues [33]. The shift in peak expression observed in both the brain and liver indicates a fundamental adaptation mechanism that may optimize metabolic processes based on predictable food availability. This adaptive flexibility could help channel catfish maintain metabolic efficiency under varying environmental conditions, ensuring better growth and survival in their natural habitats. Further studies are required to investigate how these rhythmic shifts in gene expression influence fish growth and immunity over long periods and under varying environmental conditions.

While this study provides important insights into the circadian regulation in channel catfish, the relatively short experimental duration may limit the assessment of long-term physiological adaptations, and the exclusion of other tissues beyond the brain and liver constrains a more comprehensive understanding of systemic circadian coordination, which will be explored further in future studies.

## 5. Conclusions

The results of this study provide insights into the period gene family in channel catfish, highlighting the significance of feeding rhythms in circadian regulation. By cloning and characterizing four period genes—*Ipper1b*, *Ipper2*, *Ipper2l*, and *Ipper3*—and examining their expression patterns across different tissues and feeding conditions, we demonstrated distinct expression profiles that vary by gene and tissue type. Notably, the rhythmic expression of these genes is significantly modulated by a strict feeding schedule, particularly in brain and liver tissues, which emphasizes the impact of the feeding time on circadian gene regulation. The high homology of these genes with period genes in other fish species, despite the absence of *per1a*, indicates evolutionary conservation in circadian mechanisms across species. This study not only expands the understanding of the molecular basis of circadian rhythms in channel catfish but also provides a valuable genetic framework for future investigations into the interplay between environmental cues, feeding behavior, and circadian biology in aquaculture species.

## Figures and Tables

**Table 1:** Basic information of channel catfish period family proteins. Molecular weights, isoelectric points (pI), GRAVY values, amino acid counts, and compositions of various amino acid types for the 4 period proteins of *I. punctatus*.

Properties	IpPER1b	IpPER2	IpPER2l	IpPER3
M (kDa)	154.56	143.85	149.31	138.48
pI	5.81	5.98	5.97	5.75
GRAVY	−0.573	−0.699	−0.615	−0.604
Amino acid number	1443	1313	1373	1278
Amino acid types	Percentage (%)			
Aliphatic	30.15	27.04	29.35	30.36
Aromatic	4.57	6.40	5.97	5.16
Sulphur	4.50	4.57	3.71	4.38
Basic	11.16	12.11	12.02	12.28
Acidic	19.33	21.86	22.00	20.81
Aliphatic hydroxyl	21.07	19.50	20.03	18.15
tRNA synthetase class I	40.06	41.20	40.06	41.08
tRNA synthetase class II	59.94	58.80	59.94	58.92

## Data Availability

The data supporting the reported results can be found in the Supplementary Materials attached to this manuscript.

## Supplementary Materials

The following supporting information can be downloaded at: https://www.mdpi.com/article/10.3390/cimb47060438/s1**.**

## References

[1] Mohawk J.A., Green C.B. (2012). Central and peripheral circadian clocks in mammals. Annu. Rev. Neurosci..

[2] Reppert S.M., Weaver D.R. (2002). Coordination of circadian timing in mammals. Nature.

[3] Partch C.L., Green C.B., Takahashi J.S. (2014). Molecular architecture of the mammalian circadian clock. Trends Cell Biol..

[4] Reick M., Garcia J.A. (2001). An analog of clock operative in the mammalian forebrain. Science.

[5] Gekakis N., Staknis D. (1998). Role of the CLOCK protein in the mammalian circadian mechanism. Science.

[6] Garbe D.S., Fang Y. (2013). Cooperative interaction between phosphorylation sites on PERIOD maintains circadian period in *Drosophila*. PLoS Genet..

[7] Fu L., Pelicano H. (2002). The circadian gene *Period2* plays an important role in tumor suppression and DNA damage response in vivo. Cell.

[8] Von Schantz M., Jenkins A. (2006). Evolutionary history of the vertebrate period genes. J. Mol. Evol..

[9] Wang H. (2008). Comparative analysis of period genes in teleost fish genomes. J. Mol. Evol..

[10] Chen R., Schirmer A. (2009). Rhythmic PER abundance defines a critical nodal point for negative feedback within the circadian clock mechanism. Mol. Cell.

[11] Sánchez-Vázquez F.J., López-Olmeda J.F. (2019). Environmental cycles, melatonin, and circadian control of stress response in fish. Front. Endocrinol..

[12] Nie K., Wang K. (2018). Effects of circadian clock protein Per1b on zebrafish visual functions. Chronobiol. Int..

[13] Serin I., Pehlivan S. (2023). A new clock is running for multiple myeloma: Circadian clock protein-*Period 3* (*PER-3*) polymorphism. Balk. J. Med. Genet..

[14] Guess J., Burch J.B. (2009). Circadian disruption, *Per3*, and human cytokine secretion. Integr. Cancer Ther..

[15] Bishehsari F., Voigt R.M. (2020). Circadian rhythms and the gut microbiota: From the metabolic syndrome to cancer. Nat. Rev. Endocrinol..

[16] Damiola F., Le Minh N. (2000). Restricted feeding uncouples circadian oscillators in peripheral tissues from the central pacemaker in the suprachiasmatic nucleus. Genes Dev..

[17] Wang M., Zhong Z. (2015). The zebrafish period2 protein positively regulates the circadian clock through mediation of retinoic acid receptor (RAR)-related orphan receptor α (Rorα). J. Biol. Chem..

[18] Waterhouse A., Bertoni M. (2018). SWISS-MODEL: Homology modelling of protein structures and complexes. Nucleic Acids Res..

[19] Takahashi J.S., Hong H.-K. (2008). The genetics of mammalian circadian order and disorder: Implications for physiology and disease. Nat. Rev. Genet..

[20] Dunlap J.C. (1999). Molecular bases for circadian clocks. Cell.

[21] Zeng H., Qian Z. (1996). A light-entrainment mechanism for the Drosophila circadian clock. Nature.

[22] Postlethwait J.H., Woods I.G. (2000). Zebrafish comparative genomics and the origins of vertebrate chromosomes. Genome Res..

[23] Cermakian N., Whitmore D. (2000). Asynchronous oscillations of two zebrafish CLOCK partners reveal differential clock control and function. Proc. Natl. Acad. Sci. USA.

[24] Taylor J.S., Van de Peer Y. (2001). Comparative genomics provides evidence for an ancient genome duplication event in fish. Philos. Trans. R. Soc. Lond. B Biol. Sci..

[25] Force A., Lynch M. (1999). Preservation of duplicate genes by complementary, degenerative mutations. Genetics.

[26] Schmutz J., Cannon S.B. (2010). Genome sequence of the palaeopolyploid soybean. Nature.

[27] Kaneko M., Cahill G.M. (2005). Light-dependent development of circadian gene expression in transgenic zebrafish. PLoS Biol..

[28] Velarde E., Haque R. (2009). Circadian clock genes of goldfish, *Carassius auratus*: cDNA cloning and rhythmic expression of period and cryptochrome transcripts in retina, liver, and gut. J. Biol. Rhythms.

[29] Di Rosa V., López-Olmeda J.F. (2016). Daily rhythms of the expression of key genes involved in steroidogenesis and gonadal function in zebrafish. PLoS ONE.

[30] Xu P., Wang Y. (2014). Genome sequence and genetic diversity of the common carp, Cyprinus carpio. Nat. Genet..

[31] Toloza-Villalobos J., Arroyo J.I., Opazo J.C. (2015). The circadian clock of teleost fish: A comparative analysis reveals distinct fates for duplicated genes. J. Mol. Evol..

[32] Nisembaum L.G., Velarde E. (2012). Light-dark cycle and feeding time differentially entrains the gut molecular clock of the goldfish (*Carassius auratus*). Chronobiol. Int..

[33] Sánchez-Vázquez F.J., Madrid J.A. (1996). Demand feeding and locomotor circadian rhythms in the goldfish, *Carassius auratus*: Dual and independent phasing. Physiol. Behav..

[34] Volkoff H., Rønnestad I. (2020). Effects of temperature on feeding and digestive processes in fish. Temperature.

[35] Paredes M.F., Sorrells S.F. (2016). Brain size and limits to adult neurogenesis. J. Comp. Neurol..

[36] López-Olmeda J.F., Tartaglione E.V. (2010). Feeding entrainment of food-anticipatory activity and Per1 expression in the brain and liver of zebrafish under different lighting and feeding conditions. Chronobiol. Int..

[37] Kalsbeek A., la Fleur S. (2011). Circadian disruption and SCN control of energy metabolism. FEBS Lett..

[38] Mistlberger R.E. (2009). Food-anticipatory circadian rhythms: Concepts and method. Eur. J. Neurosci..

[39] Costa L.S., Serrano I. (2016). Circadian rhythms of clock gene expression in Nile tilapia (*Oreochromis niloticus*) central and peripheral tissues: Influence of different lighting and feeding conditions. J. Comp. Physiol. B.

[40] Escobar C., Martínez-Merlos M.T., Angeles-Castellanos M., del Carmen Miñana M., Buijs R.M. (2007). Unpredictable feeding schedules unmask a system for daily resetting of behavioural and metabolic food entrainment. Eur. J. Neurosci..

[41] Davidson A.J., Castanon-Cervantes O. (2009). Visualizing jet lag in the mouse suprachiasmatic nucleus and peripheral circadian timing system. Eur. J. Neurosci..

[42] Rivera-Zavala J.B., Molina-Aguilar C. (2017). Daytime restricted feeding modifies the daily regulation of fatty acid β-oxidation and the lipoprotein profile in rats. Br. J. Nutr..

[43] Asher G., Schibler U. (2011). Crosstalk between components of circadian and metabolic cycles in mammals. Cell Metab..

[44] Sahar S., Sassone-Corsi P. (2009). Metabolism and cancer: The circadian clock connection. Nat. Rev. Cancer.

[45] Peek C.B., Levine D.C. (2017). Circadian clock interaction with *HIF1α* mediates oxygenic metabolism and anaerobic glycolysis in skeletal muscle. Cell Metab..

[46] Delaunay F., Thisse C., Thisse B., Laudet V. (2003). Differential regulation of *period 2* and *period 3* expression during development of the zebrafish circadian clock. Gene Expr. Patterns.

[47] Vera L.M., Negrini P. (2013). Light and feeding entrainment of the molecular circadian clock in a marine teleost (*Sparus aurata*). Chronobiol. Int..

[48] Hatori M., Vollmers C. (2012). Time-restricted feeding without reducing caloric intake prevents metabolic diseases in mice fed a high-fat diet. Cell Metab..

[49] Ali M., Nicieza A. (2003). Compensatory growth in fishes: A response to growth depression. Fish Fish..

