# Supplementary material for: Molecular Characterization and Feeding-Associated Expression Dynamics of the Period Gene Family in Channel Catfish (Ictalurus punctatus)
Source: Curr Issues Mol Biol. 2025 Jun 9;47(6):438. doi: 10.3390/cimb47060438 (PMC12191489; doi:10.3390/cimb47060438)
Supplement: Supplementary file 1 [file cimb-47-00438-s001.zip › Table S1.pdf]

Table S1 The sequences of primers for gene clone

Target gene	Primer sequences (5'-3')	Product (bp)
per1b F	GACCATGATTACGCCAAGCTTATGAGTGATGACA ACTCAGACTCCG	4332
per1b R	AGTGAATTCGAGCTCGGTACCTCAGTGTCTCATAT CAGTTGCATGG	
per2 F	GACCATGATTACGCCAAGCTTATGCAGAAGTCCA TCTCCATGTC	4122
per2 R	AGTGAATTCGAGCTCGGTACCTCATTGCATCTGGT CACGAGG	
per2l F	GACCATGATTACGCCAAGCTTATGTCAGGTGAGT CTGAGCTGAAG	3942
per2l R	AGTGAATTCGAGCTCGGTACCCTAGGAAACACAG TCTAATAAGACATGC	
per3 F	GACCATGATTACGCCAAGCTTATGCTCAGTGGAG ACAACTACCAG	3843
per3 R	AGTGAATTCGAGCTCGGTACCTCAGGTGTCAGAG GCGGGG	
