# Supplementary material for: Molecular Characterization and Feeding-Associated Expression Dynamics of the Period Gene Family in Channel Catfish (Ictalurus punctatus)
Source: Curr Issues Mol Biol. 2025 Jun 9;47(6):438. doi: 10.3390/cimb47060438 (PMC12191489; doi:10.3390/cimb47060438)
Supplement: Supplementary file 1 [file cimb-47-00438-s001.zip › Table S2.pdf]

Table S2 The sequences of primers for qPCR

Target gene	Primer sequences (5'-3')	Product (bp)
per1b F	CGGAGAAGCCCAGTTCATT	106
per1b R	GTCTCTGATGGGCAGTTGATAG	
per2 F	GAGCAGTCACACCAGCAATA	131
per2 R	GCAAACTCTCACTCTCCTCTAAC	
per2L F	CTGCACGAGGAATGGTGAATA	90
per2L R	CCGATGACGAAGGACACTTT	
per3 F	ACTGTGTGGACAGCATCATTAG	104
per3 R	TGTGGTAGAAGAAGAGGAGGTAG	
α-tubulin F	AGCCATACAATTCCATCCTGACC	95
α-tubulin R	GCGGCAGATGTCGTAGATGG	
