# Supplementary figures and images for: Molecular Characterization and Feeding-Associated Expression Dynamics of the Period Gene Family in Channel Catfish (Ictalurus punctatus)
Source: Curr Issues Mol Biol. 2025 Jun 9;47(6):438. doi: 10.3390/cimb47060438 (PMC12191489; doi:10.3390/cimb47060438)

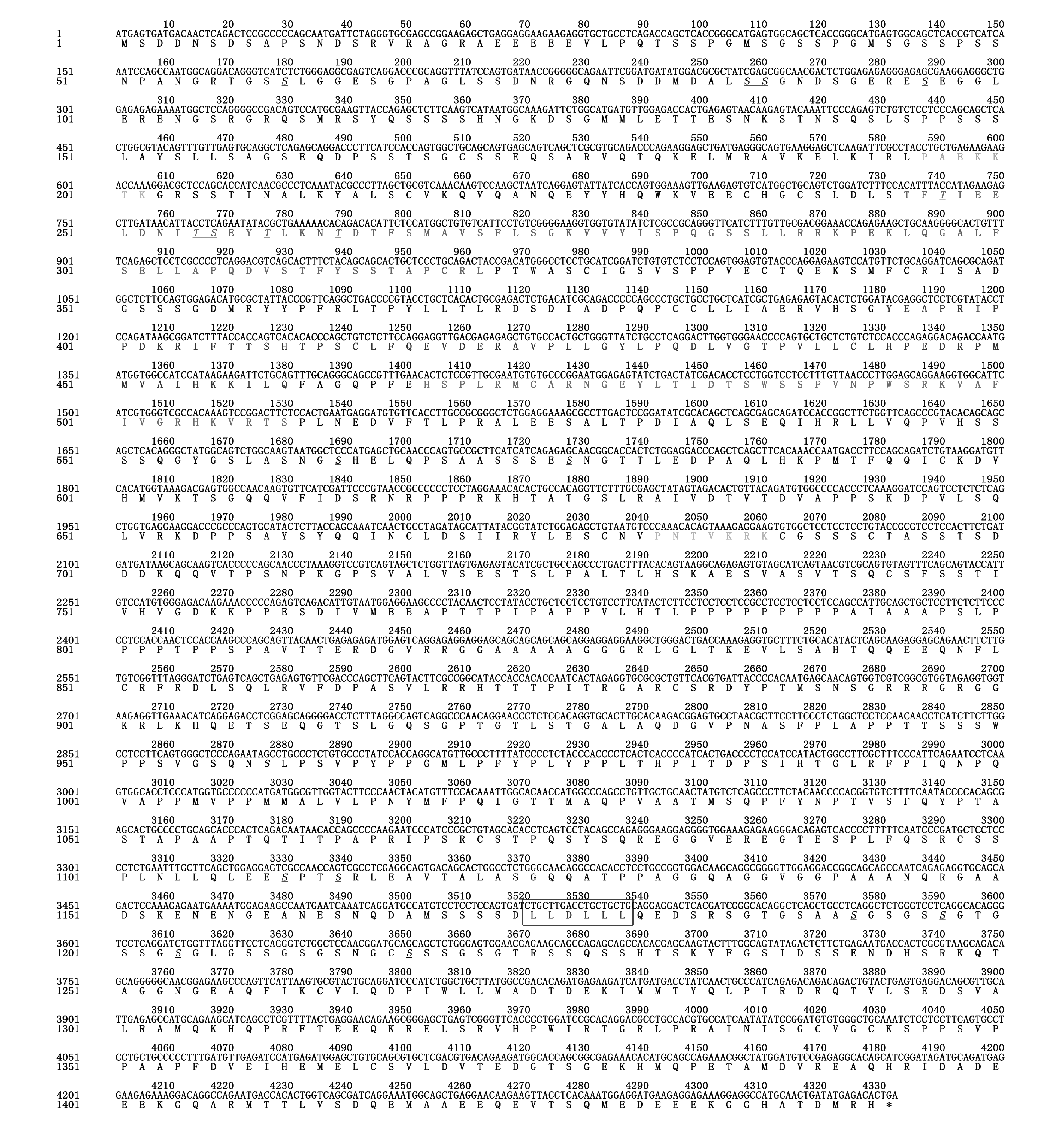

Supplement: Supplementary file 1 [file cimb-47-00438-s001.zip › figure s1.tif]

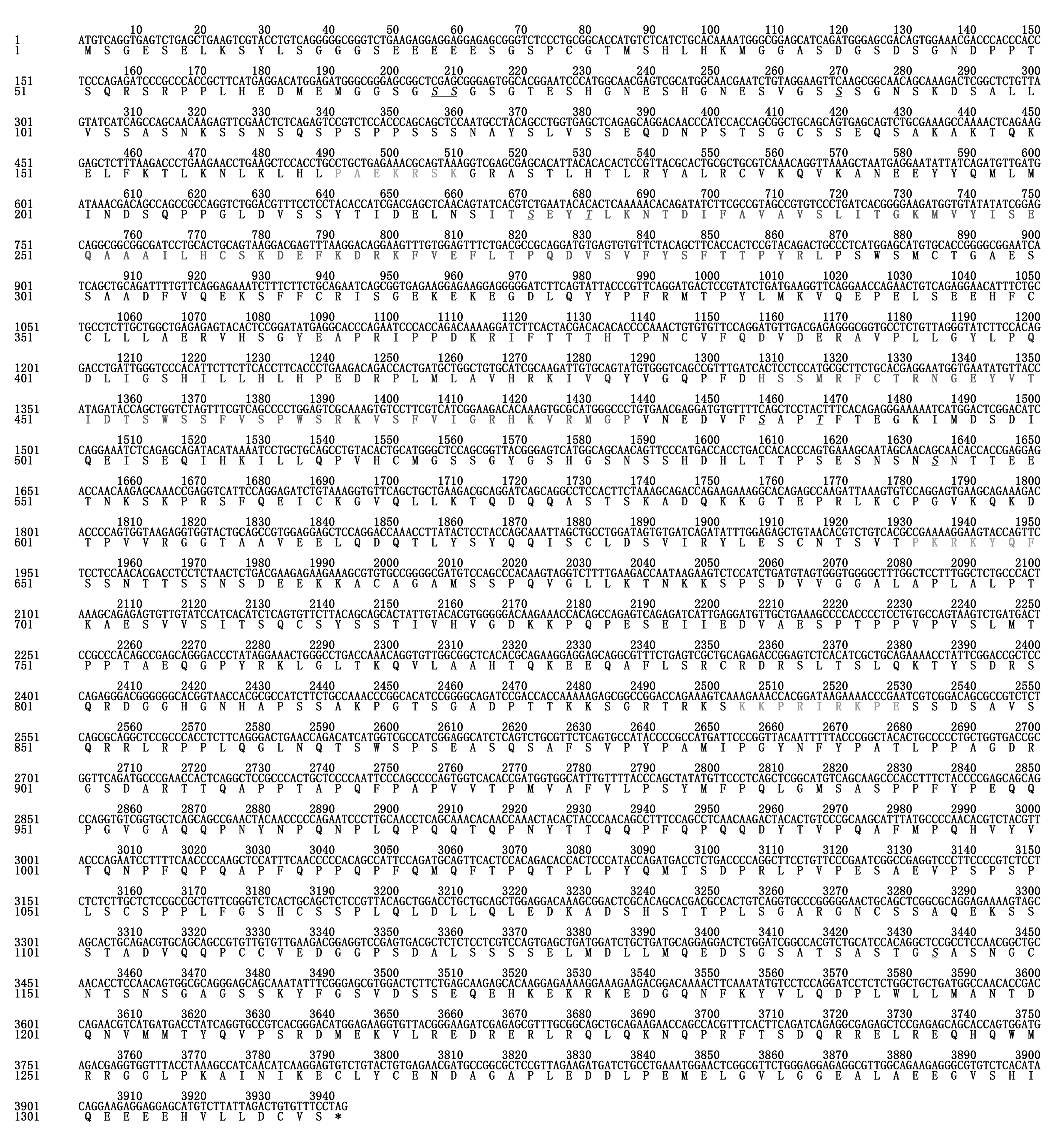

Supplement: Supplementary file 1 [file cimb-47-00438-s001.zip › figure s2.tif]

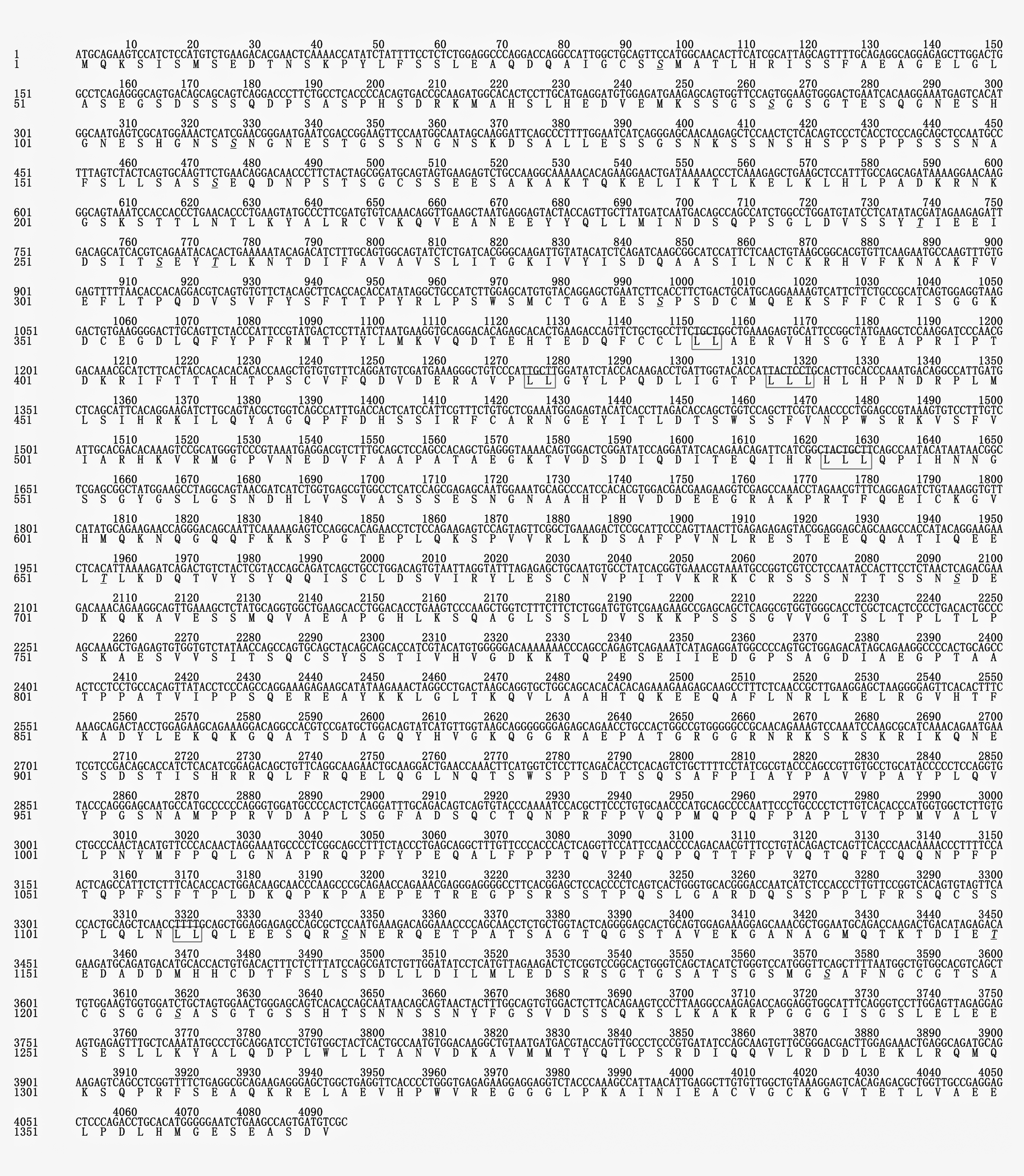

Supplement: Supplementary file 1 [file cimb-47-00438-s001.zip › figure s3.tif]

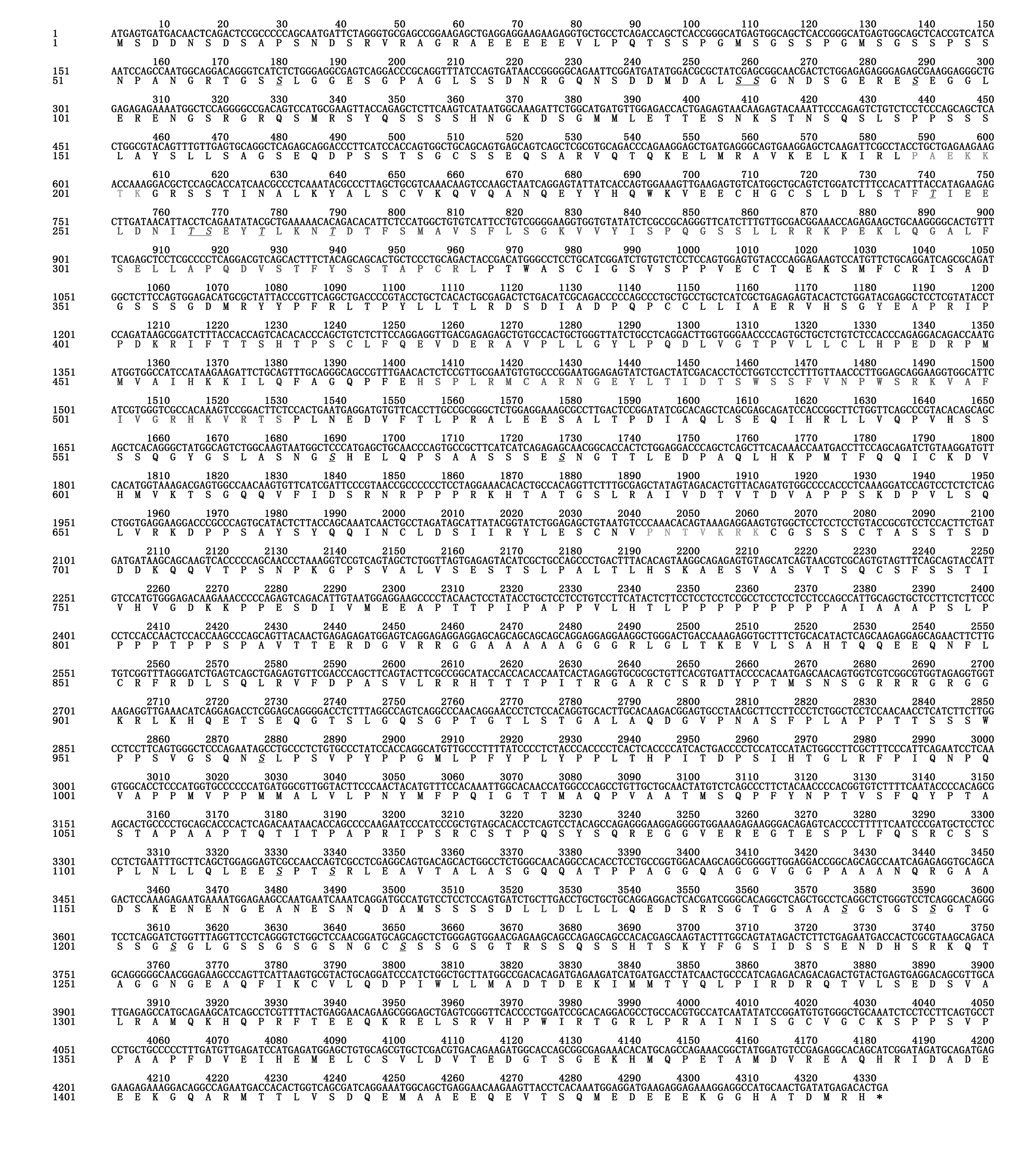

Supplement: Supplementary file 1 [file cimb-47-00438-s001.zip › figure s4.tif]
